# Complete Genome Sequences of Three Novel *Pseudomonas fluorescens* SBW25 Bacteriophages, Noxifer, Phabio, and Skulduggery

**DOI:** 10.1128/genomeA.00725-17

**Published:** 2017-08-03

**Authors:** Joanna K. Wojtus, Jess L. Fitch, Eli Christian, Tara Dalefield, Jacob K. Lawes, Kirtana Kumar, Craig L. Peebles, Eric Altermann, Heather L. Hendrickson

**Affiliations:** a Massey Phage Whānau, Institute of Natural and Mathematical Science, Massey University, Auckland, New Zealand; b School of Life Sciences, University of Glasgow, Glasgow, United Kingdom; c Department of Biological Sciences, University of Pittsburgh, Pittsburgh, Pennsylvania, USA; d AgResearch, Palmerston North, New Zealand; e Riddet Institute, Massey University, Palmerston North, New Zealand

## Abstract

Three novel bacteriophages, two of which are jumbophages, were isolated from compost in Auckland, New Zealand. Noxifer, Phabio, and Skulduggery are double-stranded DNA (dsDNA) phages with genome sizes of 278,136 bp (Noxifer), 309,157 bp (Phabio), and 62,978 bp (Skulduggery).

## GENOME ANNOUNCEMENT

*Pseudomonas fluorescens* SBW25 is a well-known model microbe for a wide range of evolutionary and environmental microbiology investigations (1–5). Previous work has reported the genome sequence of a single strictly lytic bacteriophage for this host, SBW25Φ2 (or phi2; GenBank accession no. FN594518.1, 43,144 bp) (6–9). In order to increase our understanding of the diversity of *P. fluorescens* SBW25 phages, an undergraduate class at Massey University conducted a bacteriophage hunt in the region of Auckland, New Zealand, using this strain as the host.

A total of 56 samples (soil, compost, and water) from the Auckland region were processed by direct plating on LB-Top agar (0.7%). Plaques were confirmed by spot test plating, which led to the discovery of three novel phages. Each phage underwent three rounds of purification and a 10-plate amplification to high titer; the phages were named Skulduggery, Noxifer, and Phabio. The plaques for Skulduggery were clear and ~2 mm in diameter, while the plaques for phages Noxifer and Phabio were tiny pinprick-sized plaques. Morphological characterization of these phages by transmission electron microscopy revealed large icosahedral heads and contractile tails for Noxifer (head diameter, 130.9 nm; tail length, 191.6 nm) and Phabio (head diameter, 134.0 nm; tail length, 174 nm) and a smaller capsid size and minimal tail for Skulduggery (head diameter, 72.5 nm; tail length, 30.3 nm). Phabio and Noxifer are therefore included in the *Myoviridae* family and Skulduggery in the *Podoviridae* family. Genomic DNA was extracted (phage DNA isolation kit [Norgen, CA, catalog no. 46850]) and further purified by phenol extraction and ethanol precipitation.

Purified genomic DNA was sequenced on an Illumina MiSeq 2 × 150-bp paired-end run. Sequence preprocessing was performed by Mauro Truglio (Massey University, Palmerston North, New Zealand). Sequencing reads were processed and trimmed with SolexaQA to a threshold of 0.01. Reads were assembled using Velvet, and single contigs were produced, each of which had at least 56× coverage (average coverage, 106×). For verification, nearly identical assemblies were also obtained using the MIRA assembler. Noxifer and Phabio appear to be circularly permuted, while Skulduggery appears to be linear. Genome annotation of these phages was conducted using DNA Master (J. G. Lawrence [http://cobamide2.bio.pitt.edu]), a program that integrates Glimmer 3.02, GeneMark, BLAST, and Shine-Dalgarno (SD) position-weighted scores, as well as general annotation tools. Aragorn and tRNAscan-SE were used to detect legitimate tRNAs in Phabio and Noxifer and one probable small stable RNA in Noxifer (Table 1).

**TABLE 1:** Summary of genome data for three novel *Pseudomonas* phages isolated in New Zealand

Phage	GenBank accession no.	Size (bp)	G+C content (%)	No. of ORFs (including tRNAs)^a^	No. of tRNAs	Yr of isolation
Noxifer	MF063068	278,136	53.5	338	4 (Lys, Asn, Asp, Tyr) & 1 small RNA	2013
Phabio	MF042360	309,157	43.0	471	3 (Asn, Leu, Ser)	2013
Skulduggery	MF042361	62,978	60.7	72	0	2013

^a^ ORFs, open reading frames.

Genome sequencing confirmed genome sizes consistent with the transmission electron microscopy (TEM) measurements. Skulduggery is a typical size, considering those of previously published *Pseudomonas* phages, with a genome size of 62,978 bases in length, and it bears structural gene similarity with *Erwinia pyrifoliae* phage PeP14 (GenBank accession no. NC_016767). However, Noxifer and Phabio are significantly larger and qualify as “jumbophages,” according to the definition of jumbophages being those over 200,000 bp (Table 1) (10). Their genome organization and size suggest that these are PhiKZ-like *Pseudomonas* phages (11). Finalized genomes were organized to align with the conventions of the PhiKZ-like and Pep14 genomes.

### Accession number(s).

These whole-genome sequences have been deposited in GenBank under the accession numbers as described in Table 1.
